# Remaining Useful Life Prediction Method for Bearings Based on Pruned Exact Linear Time State Segmentation and Time–Frequency Diagram

**DOI:** 10.3390/s25061950

**Published:** 2025-03-20

**Authors:** Xu Wei, Jingjing Fan, Huahua Wang, Lulu Cai

**Affiliations:** 1 School of Electtrical and Control Engineering, North China University of Technology, Beijing 100144, China; 2 Jiangsu Innovation Center of Intelligent Equipment Co., Ltd., Changzhou 213300, China

**Keywords:** bearing, PELT state segmentation, wavelet transform, time–frequency diagram, Informer, RUL

## Abstract

To improve the accuracy and robustness of bearing remaining useful life (RUL) prediction, this paper proposes a bearing RUL prediction method based on PELT state segmentation and time–frequency analysis, incorporating the Informer model for time-series modeling. First, the PELT (Pruned Exact Linear Time) algorithm is used to segment the vibration signals over the full life cycle of the bearing, accurately identifying critical degradation states and optimizing the stage division of the degradation process. Next, wavelet transform is applied to perform time–frequency analysis on the vibration signals, generating time–frequency spectrograms to comprehensively extract features in both the time and frequency domains. Finally, the extracted time–frequency features are used as input to predict the bearing RUL using the Informer model. As an efficient time-series prediction model, the Informer excels at handling long time series by leveraging a sparse self-attention mechanism to effectively capture the long-term dependencies in the signals. Experiments conducted on a publicly available dataset and comparisons with traditional methods demonstrate that the proposed method offers significant advantages in terms of prediction accuracy, computational efficiency, and robustness, making it more suitable for bearing health assessment and RUL prediction under complex working conditions.

## 1. Introduction

Bearings are critical components in mechanical systems, widely used in industries such as transportation, energy, and manufacturing, earning the title “joints of industry”. Their performance directly impacts the reliability and efficiency of equipment. Accurately predicting the remaining useful life (RUL) of bearings is crucial for ensuring stable operation, enhancing safety, optimizing maintenance schedules, and reducing the costs associated with unexpected downtime [1]. Current RUL prediction models for rolling bearings can be broadly categorized into three types: physics-based models, statistical models, and data-driven machine learning models.

Physics-based models predict RUL by employing mathematical or physical models of degradation phenomena (e.g., fatigue, wear, corrosion-induced cracking), such as the Paris–Erdogan crack propagation model [2], linear programming models [3], and S-N curve models [4]. Statistical models, on the other hand, establish RUL prediction methods by fitting monitored or historical data into stochastic coefficient or statistical process models. For instance, Zhu J combined the hidden Markov model with adaptive fault detection and addressed domain discrepancies using a multi-layer perceptron-based transfer learning method, improving reliability under different working conditions [5]. Wang et al. proposed a dynamic RUL prediction and optimal maintenance time (OMT) method based on a Gamma process model [6]. Similarly, Z. Huang et al. developed an adaptive skew-Wiener model for RUL prediction using online filtering and two-stage parameter estimation [7]. Li et al. employed adaptive first passage time (FPT) selection based on the 3σ rule and particle filtering to reduce random errors, significantly enhancing RUL prediction accuracy [8]. While effective, these models require a thorough understanding of degradation mechanisms, rely heavily on expert knowledge, and are costly to develop. Moreover, they are better suited for qualitative reasoning and require highly accurate input data, limiting their application under complex conditions [9,10,11].

Data-driven prediction models eliminate the need for expert knowledge by extracting representative features from collected signals for RUL prediction [12]. Advances in big data, AI, and sensing technologies enable real-time collection and storage of large-scale bearing operation data, offering unprecedented opportunities [13,14,15,16]. Shen Y et al. proposed a CNN-BiLSTM-AM model with an attention mechanism to process vibration signals, achieving higher accuracy and generalization [17]. Yu P et al. introduced a wind turbine bearing RUL prediction model combining CWT, DICNN, and GPR for graphical feature extraction [18]. Qi M et al. transformed vibration signals into time–frequency images and trained them using ViT, integrating STFT for fault diagnosis [19]. Yan J et al. developed CSPA–Informer, optimizing self-attention with a CSPA structure to reduce memory usage and computational complexity, improving efficiency and accuracy [20]. Liu B combined STFT preprocessing with CNN-LSTM and CBAM for an end-to-end RUL prediction method [21]. Mohammadreza Kaji trained a CAE model with health data and constructed a health index (HI) using Mahalanobis distance (MD) for RUL prediction [22]. While data-driven approaches dominate bearing RUL prediction, challenges remain in enhancing accuracy and reducing computational costs.

Rolling bearing fatigue typically progresses through three stages: normal, fault, and failure, each exhibiting distinct degradation characteristics [23]. Effective degradation stage segmentation is critical for improving RUL prediction accuracy [23,24,25]. P. Shakya achieved real-time detection of health states and damage stages of rolling bearings by processing multi-domain vibration data, fusing Mahalanobis distance, and using the Chebyshev inequality for online monitoring [26]. Alkaya addressed false alarms and data loss caused by fixed thresholds in transient states by proposing a variance-sensitive adaptive threshold (Tvsa)-based PCA fault detection method [27]. Liu L utilized a BUP time-series segmentation algorithm with linear fitting to determine multi-stage boundaries [28]. Zeng L proposed a method combining sensitive feature selection and fusion to construct health indices, enabling early fault detection and improving prediction accuracy through dynamic multi-stage models [29]. Chen Dongnan et al. identified degradation stages using Mann–Kendall tests and normalized singular value correlation coefficients [30]. Liu S et al. proposed an RUL prediction method based on a stochastic process, employing statistical process control (SPC) for stage segmentation and model switching, and used the expectation–maximization (EM) algorithm for online parameter updates [31]. However, these methods often involve high computational complexity, limiting their scalability to large datasets [32]. Inspired by the aforementioned research, this paper proposes a bearing RUL prediction method based on PELT state segmentation and time–frequency maps, with the following innovative aspects:

1. In the segmentation of the bearing degradation stage, the PELT algorithm is adopted. Compared with traditional methods (such as dynamic programming or binary search), the PELT algorithm significantly reduces computational complexity through a pruning strategy, enabling fast and accurate detection of multiple change points in time series and thereby achieving effective segmentation of feature curves.
2. Unlike previous RUL prediction approaches that only consider time-domain features, this paper utilizes wavelet transform to convert the original vibration signal into time–frequency feature maps, which are then fed into a neural network model for bearing RUL prediction.
3. The Informer model is selected for bearing life prediction. Due to its improved self-attention mechanism (ProbSparse Self-Attention) and distillation mechanism, Informer can effectively enhance the computational efficiency and prediction performance of traditional Transformer models.

## 2. Materials and Methods

**Figure 1:**
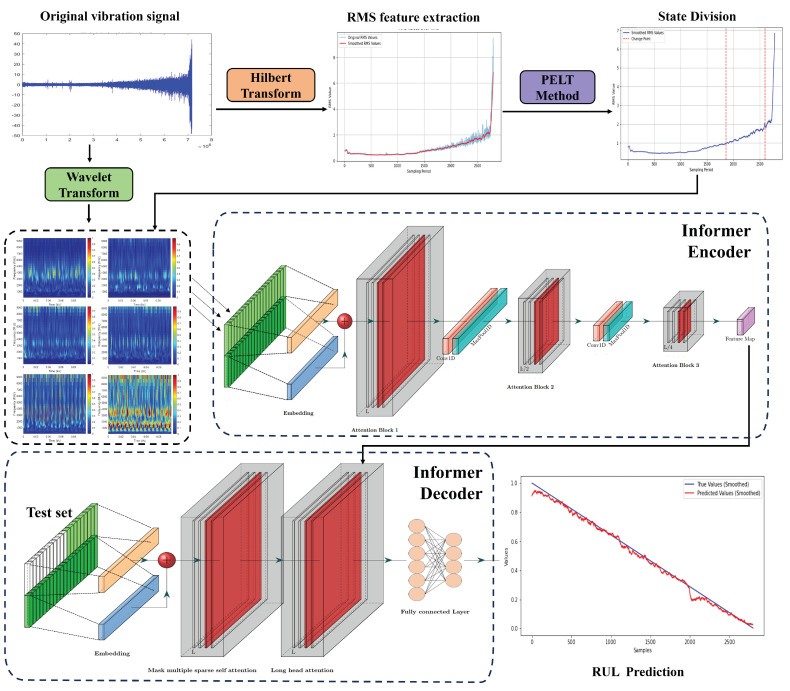
Method flowchart.

The specific process of the bearing RUL prediction method based on PELT state division and time-frequency diagram is shown in Figure 1. First, feature extraction is performed on the original vibration signal of the rolling bearing to obtain the RMS feature curve and the feature spectrum. Then, the PELT algorithm is used to monitor the change points of the smoothed RMS feature curve of the bearing vibration signal (i.e., the locations where the statistical characteristics of the data change significantly) for state division. Finally, the segmentation results are applied to the time-frequency diagram and input into the Informer bearing life prediction network for training, achieving segmented fitting and realizing a more efficient and accurate prediction of bearing RUL.

### 2.1. Feature Extraction

**Figure 2:**
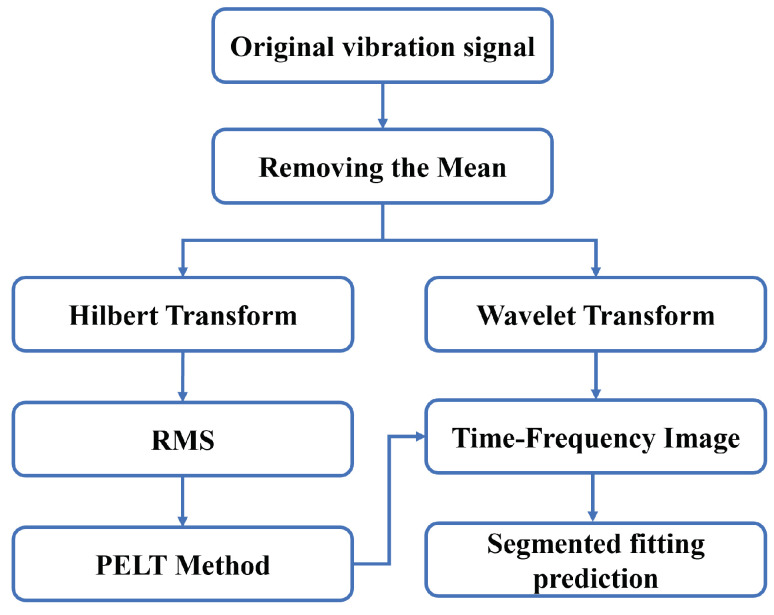
Data processing procedure.

The raw vibration signal of rolling bearings exhibits relatively stable characteristics during normal operation, with slow variations. However, this also makes RUL prediction more challenging [33]. In the evaluation of rolling bearing degradation and remaining useful life prediction, it is crucial to preprocess the raw data and select appropriate features to characterize the performance degradation trend. Degradation features can serve as health indicators to detect the onset of the degradation phase, thereby distinguishing between the healthy and faulty states of the bearing. The preprocessing process of the raw vibration signal for rolling bearings in this paper is shown in Figure 2. After mean removal, the raw vibration signal is processed in two ways: on the one hand, the Hilbert transform is applied to extract the envelope of the signal for degradation feature extraction; on the other hand, time-frequency images of the full life cycle of the bearing are obtained through wavelet transform and other methods. The degradation feature curves will be used for subsequent bearing health state classification, while the time-frequency images, classified based on the state division results, will be used for further segmented prediction of bearing life.

Vibration signals often contain a constant offset (i.e., a DC component), which does not carry actual vibration information but may affect the calculation of the signal envelope and energy. The “mean removal” method can eliminate the DC component, allowing the signal to be analyzed with a zero mean. This also prevents issues such as an artificially elevated envelope when extracting the envelope using the Hilbert transform.

Taking the time-domain degradation feature root mean square (RMS) as an example, the feature extraction process is as follows:

(1) $$x_{d}\left(n\right)=x\left(n\right)-\frac{1}{N}\sum_{N=1}^{N}x(n)$$

The envelope of the signal is extracted using the Hilbert transform:

(2) $$\hat{x}\left(n\right)=x_{d}\left(n\right)+jHx_{d}\left(n\right)$$

(3) $$H\left\{x_{d}\left(n\right)\right\}=\frac{1}{\pi }P.V.\int_{-\infty }^{\infty }\frac{x(\tau )}{n-\tau }dr$$

(4) $$Envelope\left(n\right)=\left|\hat{x}\left(n\right)\right|=\sqrt{{x_{d}\left(n\right)}^{2}+{Hx_{d}\left(n\right)}^{2}}$$

RMS is calculated as follows:

(5) $$RMS=\sqrt{\frac{1}{M}\sum_{n=1}^{M}{\left(Envelope\left(n\right)\right)}^{2}}$$

$x_{d}\left(n\right)$ is the de-meaned signal; $x\left(n\right)$ is the original signal; $\hat{x}\left(n\right)$ is the analytical signal, which contains the real and imaginary parts; $H\left\{x_{d}\left(n\right)\right\}$ is the Hilbert transform; P.V. represents the Cauchy Principal Value integral; and $Envelope\left(n\right)$ is the envelope of the signal.

The RMS is smoothed using the moving average method. For a time series $y\left[i\right]$, with a sliding window size of *N*, the moving average $x\left[i\right]$ can be expressed as follows:

(6) $$y\left[i\right]=\frac{1}{N}\sum_{j=0}^{N-1}x\left[i+j\right]$$

**Figure 3:**
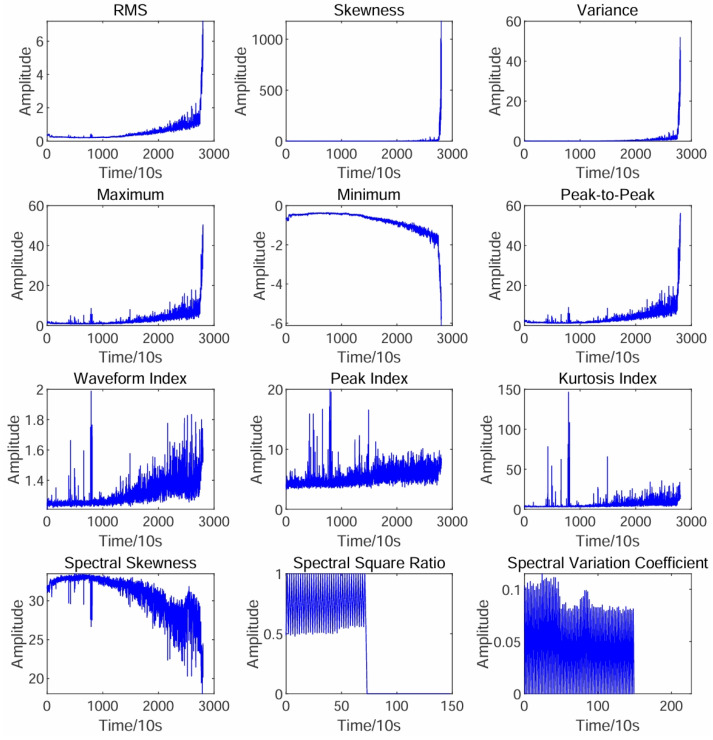
Feature curve.

In this paper, 12 time-frequency domain degradation features, including root mean square (RMS), skewness, waveform index, frequency skewness, and others, are extracted based on the signal envelope. The feature curves are shown in Figure 3. Among them, the RMS curve exhibits better monotonicity, trend correlation, and robustness compared to other feature curves, making it more suitable as a representation of the bearing degradation process.

### 2.2. Bearing Degradation State Segmentation Based on the PELT Method

After obtaining the RMS curve, the PELT (Pruned Exact Linear Time) method is applied for bearing degradation state segmentation. PELT is an efficient change-point detection algorithm widely used in time-series analysis. It can quickly identify positions where significant changes occur in statistical characteristics such as mean and variance. The method has been applied in various fields, including finance, signal processing, and bioinformatics [34,35].

Assuming that there are change points in an observation segment, the set of change points is denoted as $\tau_{1:m}=\left(\tau_{1},\ldots ,\tau_{m}\right)$. These *m* change points divide the data samples into m+1 segments, with the segment from [i:j] denoted as $y_{i:j}$. The change-point problem can be transformed into minimizing the following expression:

(7) $$\sum_{i=1}^{m}[C\left(y_{i:j}\right)+\beta ]$$

For different statistical characteristics of data samples, the chosen cost function varies accordingly. In this study, the mean is selected as the statistical characteristic, and thus the expression for the loss function is as follows:

(8) $$C\left(y_{i:j}\right)=\sum_{k=i}^{j}{\left(y_{k}-\mu_{i:j}\right)}^{2}$$

$C(y_{i:j})$ represents the cost function for the segment [i:j]; β denotes the penalty parameter, used to control the number of change points; $mu_{i:j}$ is the mean of the segment [i:j].

The dynamic programming method is used to iteratively calculate the optimal change-point positions. Let $F\left(t\right)$ represent the optimal cost function (i.e., minimized loss) up to time point *t*, and $F\left(k\right)$ represent the optimal cost function up to time point *k*. The goal of the algorithm is to find the set of change-point positions that minimizes the following expression:

(9) $$F\left(t\right)={min}_{k<t}\left(F\left(k\right)+C\left(y_{k+1:t}\right)+\beta \right)$$

The pruning strategy of the PELT algorithm aims to reduce the number of possible candidate change-point positions, thereby reducing computational complexity. This strategy is based on the following inequality:

(10) $$F\left(t\right)\leq F\left(s\right)+C\left(y_{k+1:t}\right)+\beta$$

For a candidate change point *s* and time point *t*, if the above inequality does not hold, pruning can be applied by removing the candidate change point *s*, as it cannot be part of the optimal solution.

Compared to traditional methods (such as dynamic programming or binary search), the PELT algorithm shows significant advantages when handling large-scale data, primarily in terms of balancing computational efficiency and result accuracy [36]. While the traditional dynamic programming method is accurate, it has a high computational complexity and is difficult to scale to big-data scenarios. On the other hand, the binary search method improves speed but often sacrifices accuracy. PELT, by introducing a penalty function and pruning strategy, effectively reduces redundant computations, achieving linear time complexity while still accurately detecting the change-point locations [37]. This enables PELT to perform exceptionally well in scenarios that require handling vast amounts of data. It is particularly suitable for fields such as statistical analysis, signal processing, and anomaly detection, helping users quickly identify structural changes in data. It provides more efficient and reliable analysis tools and represents a significant breakthrough in the field of stage segmentation.

### 2.3. Continuous Wavelet Transform

In this study, the time–frequency map obtained from the wavelet transform of the raw horizontal vibration signal of the bearing is used as the input for the bearing remaining useful life (RUL) prediction model. The core concept of the wavelet transform is to select a specific mother wavelet, and then generate a set of basis functions by translating and scaling it [38]. These basis functions are used to approximate the signal, thereby enabling localized time–frequency analysis of the signal. This method can capture transient changes in the signal, addressing the limitations of traditional Fourier analysis in handling transient characteristics. The continuous wavelet transform (CWT) can be expressed as follows:

(11) $$W\left(a,b\right)=\int_{-\infty }^{\infty }x\left(t\right)\frac{1}{\sqrt{\left|a\right|}}\psi^{*}\left(\frac{t-b}{a}\right)dt$$

x(t) is the input signal; ψ(t) is the mother wavelet function; *a* is the scale parameter (related to frequency); *b* is the translation parameter (related to time); and the symbol * denotes the complex conjugate. In this implementation, the “amor” wavelet from the Morlet wavelet family is selected as the mother wavelet. Its analytical expression is as follows:

(12) $$\psi \left(t\right)=\pi^{\frac{1}{4}}e^{j\omega_{0}t}e^{-\frac{t^{2}}{2}}$$

The Morlet wavelet is constructed by multiplying a sinusoidal wave with a Gaussian envelope, providing excellent time–frequency localization properties. It can simultaneously capture both local time and frequency information of the signal, making it particularly suitable for analyzing non-stationary signals. The complex form of the Morlet wavelet provides rich amplitude and phase information, which is advantageous for transient signal detection and frequency characteristic extraction. Its Gaussian envelope ensures smoothness and symmetry, reducing boundary effect interference, making it an irreplaceable tool in non-stationary signal processing [39].

### 2.4. Informer

**Figure 4:**
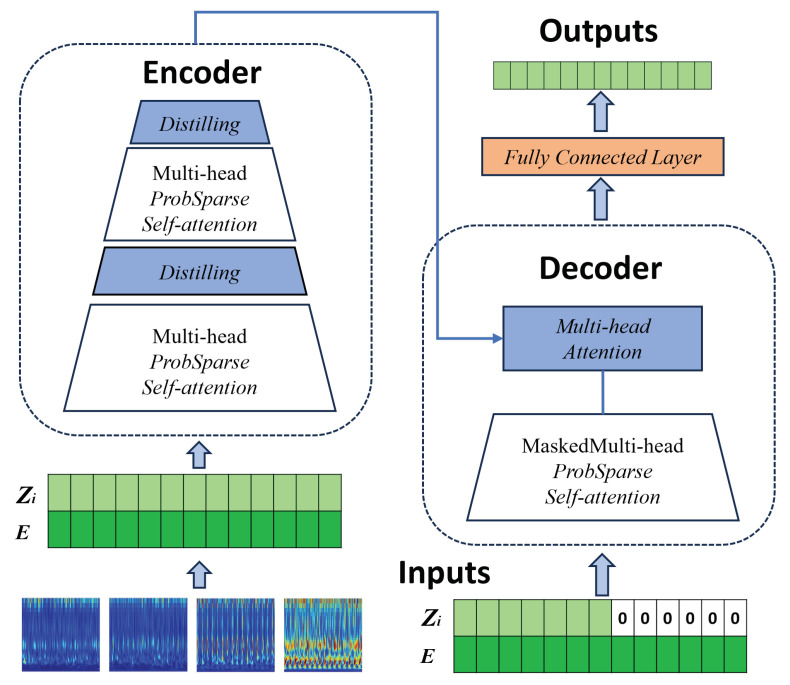
Informer model framework.

Informer is an optimized Transformer-based time-series prediction model specifically designed for handling long sequence data, making it well suited for bearing remaining useful life (RUL) prediction tasks. The framework of the Informer model is shown in Figure 4. The multi-head attention mechanism processes different feature dimensions in parallel, enabling better capture of long-term dependencies in complex time series [40]. Subsequently, the output undergoes further processing through fully connected layers to produce the final prediction [41]. The ProbSparse self-attention mechanism significantly reduces computational complexity by sparsifying attention scores, ensuring the model’s efficiency when dealing with long sequences. Additionally, the Distilling mechanism enhances the model by extracting key information and reducing redundant input, thereby maintaining both accuracy and speed.

When using time–frequency images as input for the Informer model, the image needs to be divided into *N* patches of fixed size. Each patch is then flattened into a one-dimensional vector $X_{i}$ and projected into a *d*-dimensional feature space through a learnable linear mapping *W*, resulting in the input sequence that the Informer model can process, $Z_{i}=WX_{i}+b$. However, since the Informer structure itself does not inherently capture temporal information, position encoding *E* is introduced, leading to the final network input, $Z^{\prime }=Z_{i}+E$.

Similar to Transformer, in the encoder of the Informer model, the input $X=\left(x_{1},x_{2},\ldots ,x_{n}\right)$ is mapped to a given continuous sequence $Z=\left(z_{1},z_{2},\ldots ,z_{n}\right)$, and in the decoder, the predicted results $Y=\left(y_{1},y_{2},\ldots ,y_{n}\right)$ are output. However, the attention mechanism in Transformer suffers from a sparsity issue, namely the long-tail distribution of the self-attention feature map. In this distribution, a small number of dot products contribute to the majority of the attention scores, while most pairwise dot-product calculations can be ignored [42]. The ProbSparse self-attention mechanism in the Informer model is a method that sparsifies the self-attention matrix from a probabilistic perspective, reducing computational complexity to logarithmic–linear complexity. In traditional self-attention mechanisms, the input is vectorized through an algorithm, and position and temporal information is embedded to derive three components—Query, Key, and Value—which are then used for scaled dot-product attention, as follows:

(13) $$Attention\left(Q,K,V\right)=Softmax\left(\frac{QK^{T}}{\sqrt{d_{K}}}\right)V$$

In the formula, $Q\in R^{L_{Q}\times d},K\in R^{L_{K}\times d},V\in R^{L_{V}\times d}$, and $d_{K}$ is the dimension of the input. The probabilistic form of the attention coefficient matrix for the *i*-th Query component is as follows:

(14) $$Attention\left(q_{i},K,V\right)=\sum_{j}\frac{k(q_{i},k_{j})}{\sum_{l}k(q_{i},k_{l})}v_{j}=E_{p(k_{j}|q_{i})}\left[v_{j}\right]$$

(15) $$p\left(k_{j}|q_{i}\right)=\frac{k(q_{i},k_{j})}{\sum_{l}k(q_{i},k_{l})}$$

(16) $$k\left(q_{i},k_{j}\right)=\exp\left(\frac{q_{i}k_{j}^{T}}{\sqrt{d_{K}}}\right)$$

On this basis, the KL divergence is introduced to calculate the sparsity of the Query component. The evaluation formula for the sparsity of the *i*-th Query component is as follows:

(17) $$M\left(q_{i},K\right)=ln\sum_{j=1}^{L_{k}}e^{\frac{q_{i}k_{j}^{T}}{\sqrt{d}}}-\frac{1}{L_{K}}\sum_{j=1}^{L_{K}}\frac{q_{i}k_{j}^{T}}{\sqrt{d}}$$

In the formula, the first term is the Log-Sum-Exp (LSE) calculation over all key vectors, and the second term is the arithmetic mean. By randomly selecting *u* ($u=L_{Q}\;lnL_{k}$) dot-product operations for $M\left(q_{i},K\right)$, the complexity is reduced to O(u=clnL). The largest *u* query vectors are selected, and the modified formula (13) gives the ProbSparse self-attention formula as follows:

(18) $$Attention\left(Q,K,V\right)=Softmax\left(\frac{\bar{Q}K^{T}}{\sqrt{d_{K}}}\right)V$$

In the formula, $\bar{Q}$ represents the filtered query vectors, which only include the top *u* most important query vectors. By selecting the *u* query vectors with the highest scores (u=clnL), the attention scores for each key value are computed for the top *u* query vectors. The attention scores for other query vectors are assigned as the mean of the input to the self-attention layer. By selecting the points with the highest attention scores, this approach addresses the quadratic computational complexity problem in the traditional self-attention mechanism, reducing the overall computational complexity.

As the result of the ProbSparse self-attention, the feature map in the Encoder structure is composed of many Value components. Therefore, when assigning weights to features, the distilling operation evaluates the state of all features, giving more weight to the dominant features and less weight to the others. This allows for the generation of a concentrated self-attention feature map in the next layer. The distilling operation process from layer *j* to layer j+1 is as follows:

(19) $$X_{j+1}^{t}=Maxpool\left(ELU\left(Convld\left({\left[X_{j}^{t}\right]}_{AB}\right)\right)\right)$$

In the formula, ${\left[X_{j}^{t}\right]}_{AB}$ includes the multi-head probability sparse self-attention along with its key operations in the attention block. The function Convld represents a convolution operation on the selected data along the one-dimensional time series, with the activation function being the ELU function. The self-attention distilling mechanism can reduce the length of the input data by half after each encoding step. When the Encoder module processes the input data with significantly reduced length, the required computation time is significantly reduced, and memory overhead during computation is alleviated.

The structure of the Decoder module consists of two multi-head attention layers, and the structure of both attention layers is identical. The input vector to the Decoder module is as follows:

(20) $$X_{feed-de}^{t}=Convld\left(X_{token}^{t}\;,X_{O}^{t}\right)\in R^{\left(L_{token}+L_{y}\right)\times d_{model}}$$

In the process of calculating the ProbSparse self-attention, a hidden multi-head attention mechanism is introduced to prevent all positions from attending to the next computation position. This avoids the issue of local autoregression. Finally, during the output of the encoder, the results are passed through a fully connected layer for integration, and the output dimension is consistent with the prediction method.

## 3. Experiments and Work

### 3.1. Dataset Introduction

This study uses the IEEE PHM Challenge 2012 bearing dataset, which contains vibration signals of bearings collected under laboratory conditions, recording the entire process from the beginning of operation to the occurrence of failure [43]. As shown in the Table 1, the dataset considers three operating conditions, with acceleration measurements taken every 10 s at a sampling period of 0.1 s, yielding 2560 samples with a sampling frequency of 25.6 kHz. Each operating condition provides two full-life-cycle bearing vibration signals for training the remaining useful life (RUL) prediction model, along with several test sets.

### 3.2. Feature Extraction

#### 3.2.1. RMS Feature Extraction

**Figure 5:**
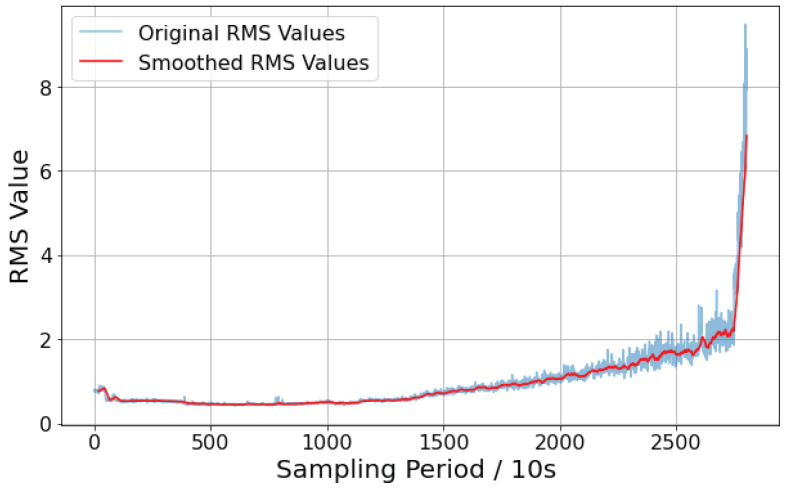
RMS plot.

As shown in Figure 5, the root mean square (RMS) exhibits a clear trend over time. With increasing degradation, the RMS value significantly increases, demonstrating good robustness and monotonicity, making it suitable for dividing the bearing degradation stages. Additionally, this metric does not require preprocessing of the vibration signal; the collected vibration signal can be directly analyzed, ensuring that no operational information of the bearing is lost.

#### 3.2.2. Time–Frequency Image Feature Extraction

**Figure 6:**
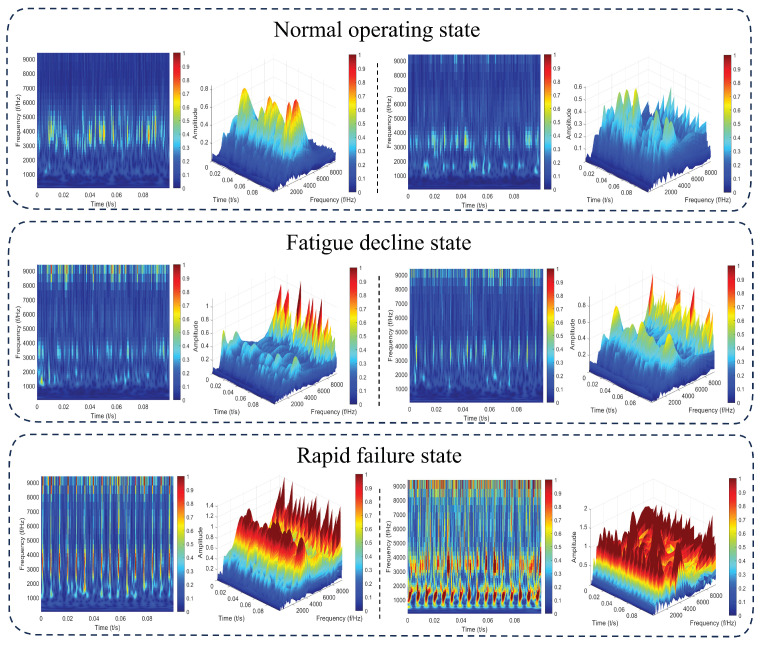
The trend of wavelet power spectrum changes over the entire lifespan of Bearing 2-2.

Taking the time–frequency image of the degradation process of Bearing2-2 as an example, we set the sampling rate to 25,600 Hz and perform the amor complex Morlet continuous wavelet transform on the vibration acceleration signal. The time axis step is 1/25,600 seconds, with a jet colormap, a color scale range of [0,1], a (0,90) view angle, a default resolution, and results saved as PNG. In the wavelet power spectrum, the horizontal axis and vertical axis represent time and frequency, respectively. The color of each point indicates the magnitude of the wavelet coefficients on the time–frequency grid, with changes in color reflecting variations in energy levels. Reddish-brown colors signify high energy levels. As shown in Figure 6, during the initial operation of the bearing, the bearing often needs to “break in” with its operating conditions, leading to a certain energy enrichment in the mid-frequency region. Subsequently, the operating state remains relatively stable, with no significant characteristics in energy distribution. As the degradation level increases further, energy explosions begin to appear in the low- and high-frequency regions, forming a “dual-peak structure”. Particularly, when the degradation level reaches 100%, high energy manifests in the image features as large areas of red, indicating regular impacts. The degree of fault is calculated as a percentage of the operating time over the entire lifespan of the bearing.

**Figure 7:**
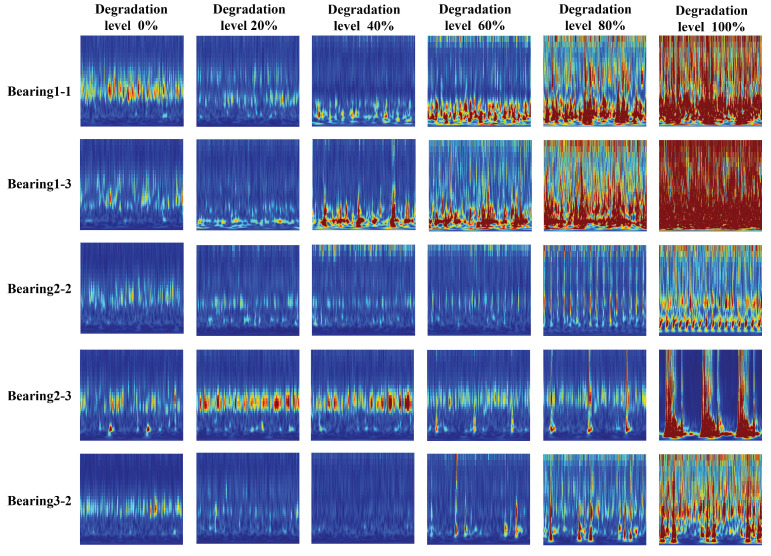
The trend of wavelet power spectrum changes over the entire lifespan of bearing degradation.

Figure 7 shows the image features of the acceleration sensor signals of several typical bearings over the entire lifespan. Clearly, the energy distribution of the vibration signals in the time–frequency domain changes with the occurrence of faults, and the image features clearly display the degradation process of all the bearings.

### 3.3. Bearing Degradation State Classification Based on the PELT Method

**Figure 8:**
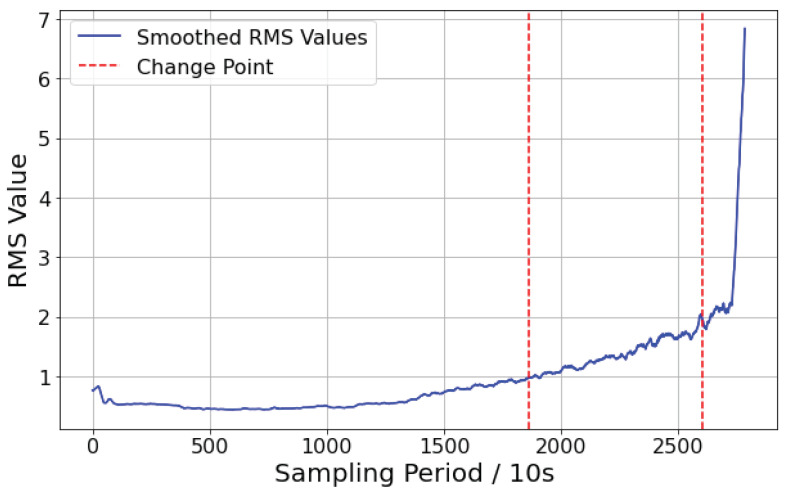
RMS segmentation image.

The bearing stage classification results based on RMS are shown in Figure 8. The bearing degradation stages have been successfully classified automatically, corresponding to the three stages of normal operation, fatigue degradation, and rapid failure in the actual bearing degradation process. The results will provide the basis for piecewise fitting in the subsequent bearing life model.

### 3.4. Bearing RUL Prediction Based on Informer

This paper selects three models—Transformer, Informer, and Informer based on PELT result segmentation fitting—to compare the prediction results on the Bearing1-1, Bearing1-3, Bearing2-2, Bearing2-3, Bearing3-2, and Bearing3-3 bearing datasets. The network model is implemented using Python 3.7, and the testing is conducted on a server equipped with an RTX-3060 GPU (NVIDIA Corporation, Santa Clara, CA, USA). and a 12th generation Intel Core i7-12700H 2.70 GHz CPU (Intel Corporation, Santa Clara, CA, USA). The input sequence length is set to 100, prediction length to 20, hidden layer dimension to 256, number of attention heads to 8, number of layers to 6 for both the encoder and decoder, attention dimension to 128, dropout rate to 0.2, batch size to 32, learning rate to 1 $\times \;10^{-4}$, and the optimizer used is AdamW.

The Mean Absolute Error (MAE) is used to describe the average absolute error between the actual and predicted life; the Mean Squared Error (MSE) is used to describe the variation and accuracy between the actual and predicted life; the Root Mean Squared Error (RMSE) is used to describe the accuracy of the actual and predicted life. The smaller the calculated values of these three evaluation metrics, the better the model’s prediction results align with the real outcomes, indicating better prediction performance. The calculation formulas for each indicator are as follows:

(21) $$MAE=\frac{1}{n}\sum_{i=1}^{n}\left|{\hat{y}}_{i}-y_{i}\right|$$

(22) $$MSE=\frac{1}{n}\sum_{i=1}^{n}{\left({\hat{y}}_{i}-y_{i}\right)}^{2}$$

(23) $$RMSE=\sqrt{\frac{1}{n}\sum_{i=1}^{n}{\left({\hat{y}}_{i}-y_{i}\right)}^{2}}$$

In the formula, *n* is the number of test data samples, $y_{i}$ is the actual life value of the test data, and ${\hat{y}}_{i}$ is the predicted life based on the state classification and time–frequency domain features of the deep learning bearing life prediction method. Additionally, the C-index is used to assess the ranking ability of the model in terms of predicting the life of bearings. The C-index is calculated as follows:

(24) $$C=\frac{1}{N_{\mathrm{pairs}}}\sum_{i\neq j}I\left(\mathrm{sign}\left({\hat{y}}_{i}-{\hat{y}}_{j}\right)=\mathrm{sign}\left(y_{i}-y_{j}\right)\right)$$

where

- $N_{\mathrm{pairs}}$ is the total number of pairs of samples;
- I(·) is the indicator function, which returns 1 if the condition inside is true, and 0 otherwise;
- sign(x) is the sign function, which returns 1 if x>0, −1 if x<0, and 0 if x=0.

To eliminate the impact of randomness, the prediction experiments for the six sets of bearing data will be repeated 20 times, and the final average value will be taken as the output result of the indicator.

**Figure 9:**
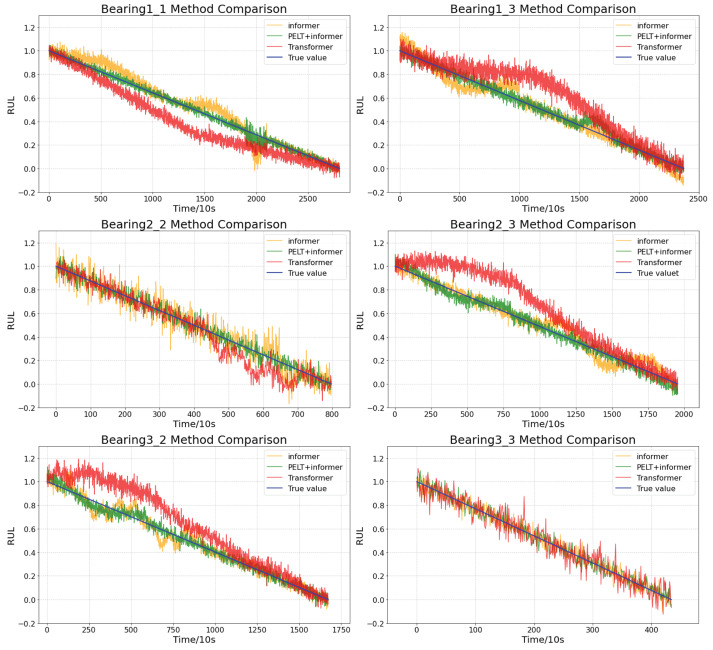
Dataset prediction results.

As shown in Table 2, the Informer prediction method with segmented fitting based on PELT results significantly outperforms the traditional Informer and Transformer methods in prediction accuracy evaluation metrics such as MAE, MSE, RMSE, and C-index. From observing Figure 9, it can be seen that when dealing with shorter time-series problems (e.g., Bearing2-2, Bearing3-3), the prediction performance of the three methods is quite similar. However, when handling longer time-series prediction problems, the Informer prediction method based on PELT segmented fitting, due to its superior feature capturing ability and the segmented fitting characteristic, produces prediction outputs that are more closely aligned with the actual values, with fewer instances of sharp disturbances.

**Figure 10:**
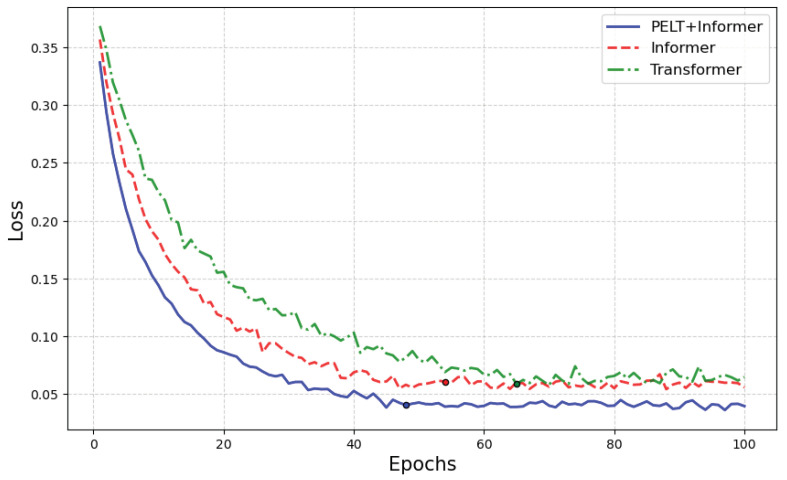
Loss curve.

Additionally, as shown in Figure 10, the Informer model, which performs segmented life prediction based on the PELT bearing degradation state division results, has a smaller loss value between the predicted and actual values compared to the other two models. It also achieves convergence with fewer iterations (the “dots” in Figure 10 indicate the points where the model’s predictions no longer show significant changes as training progresses), demonstrating higher computational efficiency and the potential to save computational resources to some extent.

## 4. Conclusions

The time–frequency characteristics of bearing vibration signals can effectively reflect their operating conditions and fault features. Features in different time–frequency domains, such as amplitude, frequency components, and energy distribution, are directly related to the degradation level of the bearing. By extracting time–frequency features associated with faults, the degradation trend of bearing performance can be captured, providing a crucial basis for predicting the remaining useful life (RUL) of the bearing.

Based on the above, this paper proposes a bearing RUL prediction method based on PELT state segmentation and time–frequency images and conducts experimental verification. The main conclusions are as follows:

1. The transformation of original bearing vibration signals into two-dimensional images through continuous wavelet transform (CWT) provides an effective visualization of the bearing degradation process. As the bearing deterioration advances, both energy impacts and bursts exhibit a marked increase. Notably, the energy distribution within the low-frequency region demonstrates more substantial variations compared to the medium- and high-frequency regions, accompanied by more pronounced impact characteristics, which warrants particular attention.
2. The PELT algorithm can effectively segment the degradation stages of bearings based on the root mean square (RMS) value, providing a basis for piecewise fitting in the model network and improving the accuracy of predictions.
3. The Informer network inherits the excellent feature extraction capabilities of the Transformer in time-series forecasting while using a sparse attention mechanism to reduce computational complexity. It demonstrates superior accuracy when handling long time-series bearing datasets, improving prediction accuracy by approximately 15.83% and computational efficiency by about 30.88%.

Although the PELT algorithm enables the segmentation of bearing degradation states based on the RMS curve, parameter tuning still requires considerable manual intervention. Additionally, while the Informer framework reduces computational complexity and improves efficiency, it still demands high memory consumption during model training. Further innovative methods are needed to address memory-related challenges.

## Figures and Tables

**Table 1:** IEEE PHM Challenge 2012 bearing dataset.

Operating Conditions	Radial Force (N)	Rotational Speed (r/min)	Training Set	Test Set
Operating Condition 1	4000	1800	Bearing1-1 Bearing1-2	Bearing1-3 Bearing1-4 Bearing1-5 Bearing1-6 Bearing1-7
Operating Condition 2	4200	1650	Bearing2-1 Bearing2-2	Bearing2-3 Bearing2-4 Bearing2-5 Bearing2-6 Bearing2-7
Operating Condition 3	5000	1500	Bearing3-1 Bearing3-2	Bearing3-3

**Table 2:** Accuracy evaluation of different models in testing.

RUL Prediction Method	MAE	MES	RMSE	C-Index
informer	0.0649	0.0068	0.0827	0.9175
Transformer	0.0670	0.0079	0.0888	0.9297
PELT+informer	0.0403	0.0025	0.0501	0.9452

## Data Availability

The data supporting the findings of this study are available upon reasonable request.
